# Commentary: Bread Wheat With High Salinity and Sodicity Tolerance

**DOI:** 10.3389/fpls.2020.01194

**Published:** 2020-08-04

**Authors:** Glenn K. McDonald, Ehsan Tavakkoli, Pichu Rengasamy

**Affiliations:** ^1^ School of Agriculture, Food and Wine, University of Adelaide, Adelaide, SA, Australia; ^2^ New South Wales Department of Primary Industries, Wagga Wagga Agricultural Institute, Wagga Wagga, NSW, Australia

**Keywords:** screening, salinity, germplasm, sodium, tolerance, net dispersive charge

## Introduction

Salinity and sodicity are important soil constraints to yield. A considerable amount of effort has been expended in evaluating germplasm to improve salinity tolerance in wheat based on responses to NaCl, but developing high-yielding, salt-tolerant germplasm using physiological screening has been elusive (Bui, 2013; Genc et al., 2019). Improvements in tolerance under controlled conditions have not generally been translated to consistently high yields in the field. While salinity and sodicity can occur together, not all sodic soils are saline because the two traits are controlled by different soil processes (Stawn et al., 2015). Sodicity is related to the dispersion of clay in soil which affects soil strength and soil porosity whereas salinity is related to the concentration of soluble ions which induces osmotic stress and ion toxicity. Despite its importance, there has been relatively little work that has assessed wheat germplasm for tolerance to sodicity.

Screening for sodicity tolerance is arguably more difficult than screening for salinity tolerance because sodic soils have both physical and chemical constraints to plant growth. Sodic soils vary considerable in pH, bulk density, porosity and salinity (Naidu and Rengasamy, 1993; Rengasamy, 2010). Phenotyping for soil constraints can be done under controlled conditions, but to be relevant, screening should replicate the effects of the conditions in which the plants grow in the field (Sadras, 2019). Therefore, when evaluating germplasm for improved tolerance to sodicity it is important to understand the properties of sodic soils and use methods that mimic the constraints of sodic soils. While solution-based systems have been used, it is considered that soil-based methods are preferable (Bromham et al., 2013).

Rengasamy (2016) recently reviewed the chemistry of saline and sodic soils and highlighted the complex interactions that occur between ionic composition and ion speciation in soil and their influences on soil pH, nutrient toxicities, and structural stability. He concluded that a poor understanding of the chemistry of saline and sodic soils is one possible reason for the slow progress in improving tolerance to salinity and sodicity.

Methods of screening for improved tolerance to sodicity in controlled conditions are needed but these should consider the complex nature of sodic soils. Genc et al. (2016, 2019) recently proposed a method based on Na humate to select for tolerance to sodicity. However, their approach misinterprets sodicity and fails to distinguish sodicity effects (*via* soil physical and chemical properties) and salinity effects (osmotic and ionic effects).

## Soil Sodicity and Consequences for Screening Germplasm

Sodicity occurs when the amount of Na on the exchange complex of clay particles increases to a level where reactions with water molecules cause swelling of clay. This leads to degradation of soil structure (dispersion), low porosity and often high bulk density which in turn reduces plant available water in the soil. Clay dispersion is the key characteristic of all sodic soils.

**Figure 1:**
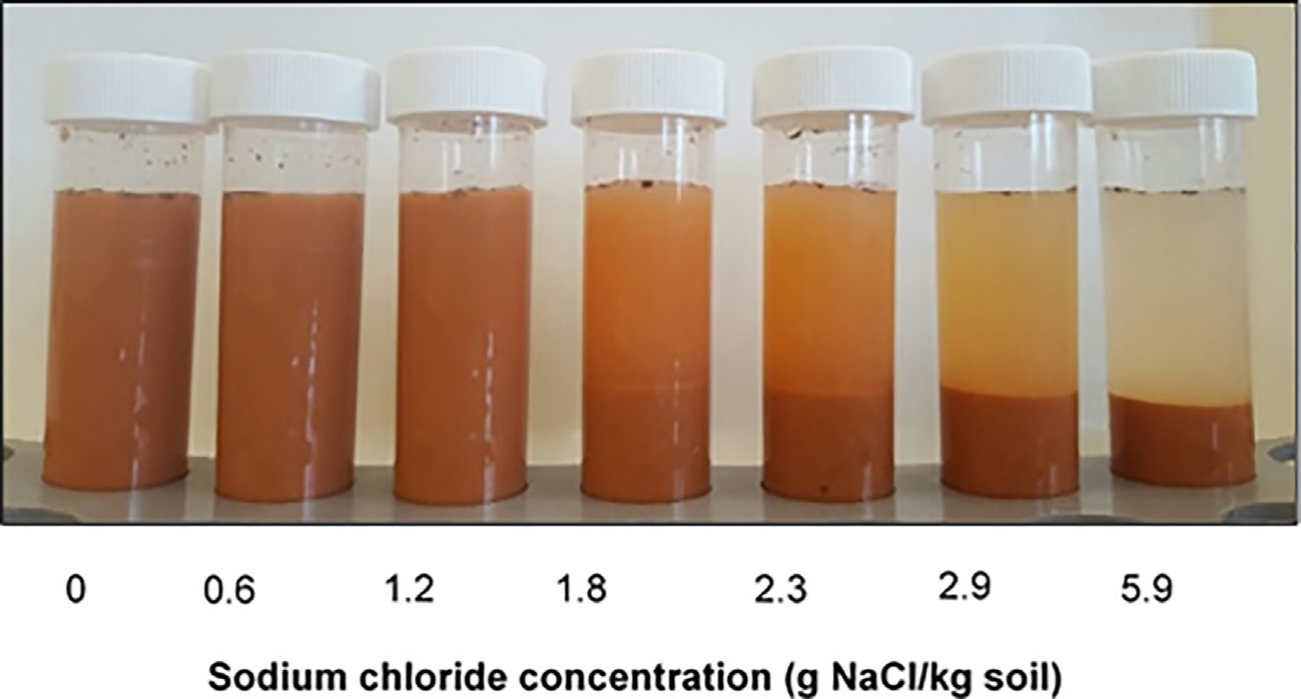
The effect of added NaCl on dispersion in a sodic soil. The soil is sodic based on its ESP but the change in NaCl concentration alters the net dispersive charge.

Soil dispersion is determined by two competing processes. Dispersive forces are influenced by the ionicity of cations on the exchange complex which is opposed by the flocculating power of the ions in the soil solution. The balance between these two forces determines whether a soil disperses, a concept described as the net dispersive charge (Rengasamy et al., 2016). Consequently, a soil defined as sodic based on its exchangeable sodium percentage (ESP) may be non-dispersive if the flocculating charge is greater than the dispersive charge (**Figure 1**).

Salinity stress is determined by the concentration and composition of dissolved ions in the soil solution which influences osmotic stress and ion toxicity. On the other hand sodicity reflects the composition of the exchangeable cations on the clay minerals in equilibrium with the soil solution, which affects the soil’s structural stability, porosity and soil strength, as well as pH and some ionic imbalances. Salinity and sodicity are fundamentally different properties of soil, but they are connected because the equilibrium between the solid phase of the soil and the ions in the soil solution determines the net dispersive charge.

The yield of crops in sodic soils can be limited by a number of different chemical and physical stresses. Consequently, it is perhaps not surprising that the importance of individual soil constraints to yield can vary considerable over sites and seasons (McDonald et al., 2012). Focussing on one trait to improve adaptation to sodic soils may not improve yields in the field unless that the trait is strongly correlated with yield.

## The Limitations of Using Na Humate to Evaluate Tolerance to Sodicity

Comparing germplasm using NaCl and Na humate allows the tolerance to high concentrations of Na and Cl to be separated (Genc et al., 2016; Genc et al., 2019) because of the low concentration of Cl in Na humate. Genc et al. (2016, 2019) also argued that using Na humate screens for sodicity tolerance because the high Na concentration of sodic soils is the main cause of reductions in plant growth. It is true that sodicity is associated with a high proportion of exchangeable Na, but to argue that reductions in growth in sodic soils are due only to Na toxicity and to equate sodicity tolerance solely to Na exclusion ignores other properties of sodic soils, such as high pH and high bulk density, that may also restrict growth. Moreover, some sodic soils have high concentrations of chloride (Dang et al., 2010), which is inconsistent with the concept of sodicity inferred by the use of Na humate. The responses of plants to Na will be affected by the concentration of Na in the soil solution not the proportion of Na on the exchange complex (the ESP); high Na will be important in a saline-sodic soil but not in all sodic soils. The use of Na humate separates Na and Cl toxicity, and in this respect it is an important new approach, but to suggest that it screens for sodicity tolerance *per se* is incorrect.

Another limitation to this method is the use of University of California (UC) potting mix, consisting of coarse sand and peat moss (Genc et al., 2016). This potting mix does not replicate the properties of sodic soils. Sodicity depends on the interaction between the ionic composition of the soil solution and the cation exchange complex of the soil because this determines the degree of dispersion on wetting, a critical characteristic of sodic soils (Rengasamy, 2016). This interaction does not occur with coarse sand. A soil’s cation exchange capacity (CEC) is determined by the negative charge on the solid matrix of the soil, which is affected by the amount and type of clay and organic matter in the soil. Sand has a very low CEC (pure sand has zero CEC) and the cation exchange processes that occur in a sodic soil do not occur in sand. The characteristic feature of a sodic soil is that it is dispersive and this cannot occur in sand. The CEC of the UC mix is due to the inclusion of peat and as a consequence the ESP of the potting mix cannot be directly compared to the ESP of a sodic soil.

Using Na humate simply screens for a specific type of salinity stress, one dominated by Na rather than Na and Cl. However, this raises a broader issue; it highlights the weakness of defining sodicity using a threshold ESP and the need to reconsider sodicity based more strongly on the principles of soil chemistry. To screen for sodicity tolerance under controlled conditions, the growth medium needs to reflect the chemical and physical properties of sodic soil, which is not achieved with the Na humate system in UC potting mix described by Genc et al. (2016, 2019).

## Improvements in Screening for Sodicity Tolerance

Screening methods need to distinguish between “sodicity” which causes physical constraints and some ionic imbalances, “Na salinity”, induced by Na humate and “NaCl salinity” where growth is affected by the ionic and osmotic effects of Na and Cl. A sodic soil has multiple physical and chemical constraints and screening for sodicity tolerance needs to reflect these constraints. The potential importance of assessing multiple traits to improve the outcome of screening programs and to understand adaptation to saline and alkaline soils has been discussed previously (Bromham et al., 2013; Bui, 2013).

Recognizing that multiple stresses may reduce growth, two approaches can be taken. First, assessing tolerance to the individual constraints of sodic soils—including high Na and Cl—to identify germplasm for a breeding program that combines tolerance to the individual stresses or second to screen for sodicity in soil using a naturally-sodic field soil or developing a synthetic soil that has similar chemical and physical properties to common types of sodic soils. Such as soil could be a non-sodic field soil or a combination of mineral clays, sand and organic matter amended with salts such as sodium carbonate to induce clay dispersion and mimic the characteristics of a sodic soil.

## Author Contributions

All authors contributed to the article and approved the submitted version.

## Conflict of Interest

The authors declare that the research was conducted in the absence of any commercial or financial relationships that could be construed as a potential conflict of interest.
